# Supplementary material for: mGluR5-mediated astrocytes hyperactivity in the anterior cingulate cortex contributes to neuropathic pain in male mice
Source: Commun Biol. 2025 Feb 20;8:266. doi: 10.1038/s42003-025-07733-5 (PMC11842833; doi:10.1038/s42003-025-07733-5)
Supplement: Supplementary file 2 — Supplementary information [file 42003_2025_7733_MOESM2_ESM.pdf]

mGluR5-mediated astrocytes hyperactivity in the anterior cingulate cortex contributes to neuropathic pain in male mice

Weida Shen[1, 2, 6, *], Fujian Chen[1, 6], Yejiao Tang[2, 3, 6], Yulu Zhao[2, 6], Linjing Zhu [2, 6], Liyang Xiang[4], Li Ning[5], Wen Zhou[2], Yiran Chen[2], Liangxue Wang[1], Jing Li[1], Hui Huang[1], Ling-Hui Zeng[1, 2, *]

[1] Anji People's Hospital, Affiliated Anji Hospital, School of Medicine, Hangzhou City University, Hangzhou, 310015, China

[2] Key Laboratory of Novel Targets and Drug Study for Neural Repair of Zhejiang Province, School of Medicine, Hangzhou City University, Hangzhou, 310015, China

[3] Institute of Pharmacology & Toxicology, College of Pharmaceutical Sciences, Key Laboratory of Medical Neurobiology of the Ministry of Health of China, Zhejiang University, Hangzhou, 310058, China

[4] School of Medicine, Nankai University, Tianjin, 300071, China

[5] Department of Anesthesiology and Surgical Intensive Care Unit, Xinhua Hospital, Shanghai Jiaotong University School of Medicine, Shanghai, 200092, China.

[6] These authors contributed equally.

[*]Corresponding authors: Ling-Hui Zeng (zenglh@hzcu.edu.cn), Weida Shen (shenwd@hzcu.edu.cn)

a

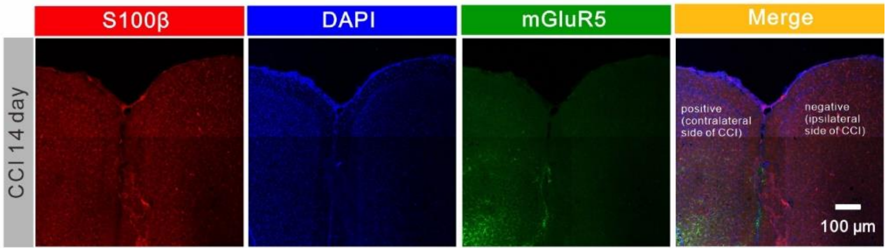

Supplementary Figure 1: Immunohistochemical images of astrocytes and mGluR5 in the ACC of CCI mice, comparing the contralateral and ipsilateral sides using the same mGluR5 antibody.

(a) Immunohistochemical images showing mGluR5 expression in astrocytes on the contralateral side of CCI, while the ipsilateral side shows no detectable expression. Red indicates s100β, green indicates mGluR5, and blue represents nuclei.

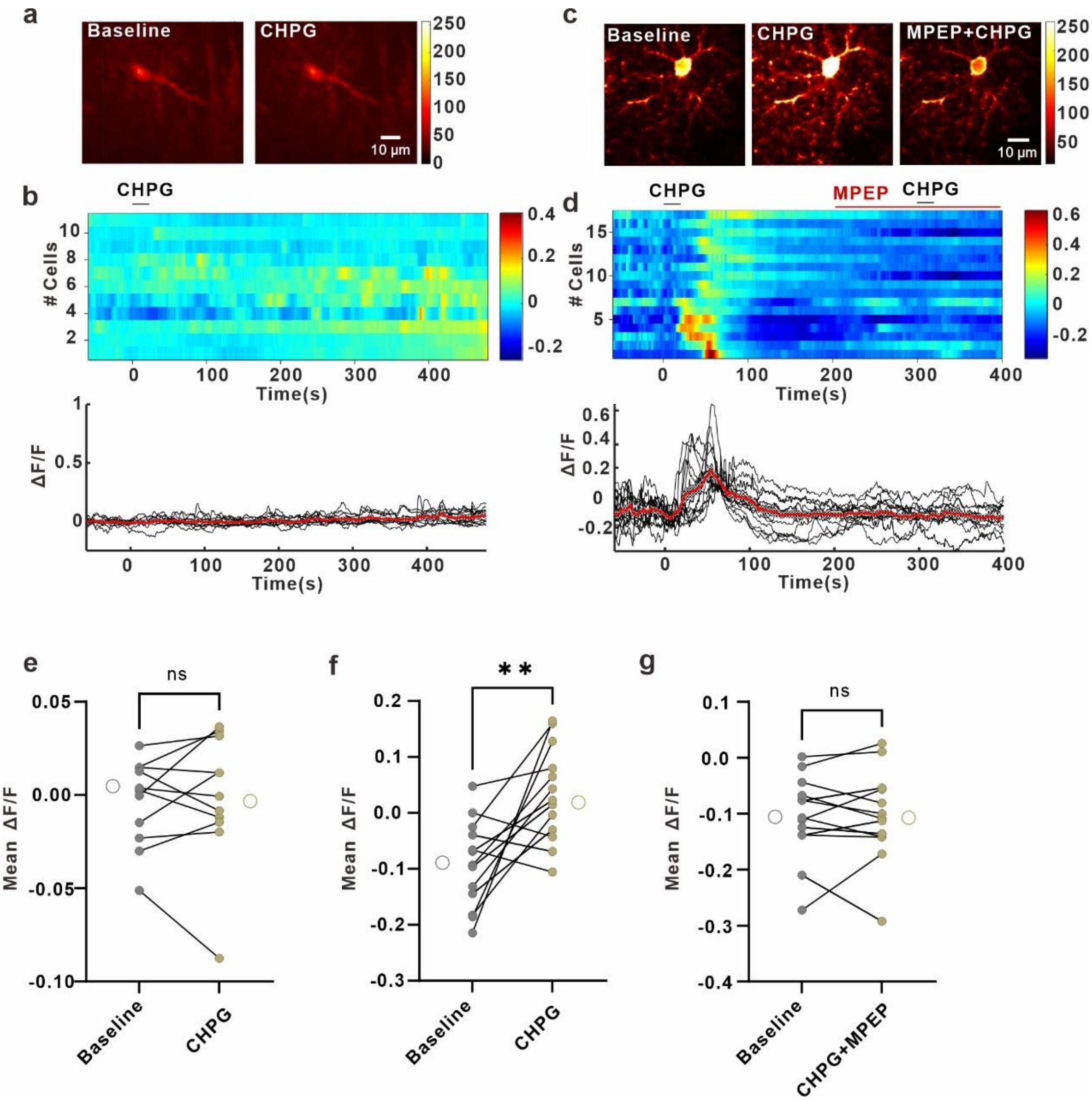

Supplementary Figure 2: CHPG-evoked Ca^{2+} signals in the ACC of neuropathic mice were blocked by the mGluR5 antagonist MPEP.

(a) Images depict a $GCaMP7b^{+}$ astrocyte before (left) and after (right) the application of CHPG (500 μM) in sham mice.

(b) Kymographs and △F/F traces of cells show no Ca^{2+} signals evoked by bath application of CHPG in $GCaMP7b^{+}$ astrocytes from sham mice, in the presence of TTX and picrotoxin. The red line represents the mean and the shading represents ± SEM.

(e) Summary plots illustrating that CHPG has no significant effects on Ca^{2+}

signals in astrocytes **(n=11 cells from 3 mice; baseline (gray circle) = 0.002543 △F/F, CHPG (brownish green circle) = -0.0007383 △F/F, *p*=0.7038, Paired t-test)**. The empty circles represent the median.

(c) Images depict a GCaMP7b^{+} astrocyte before (left) and after (right) the application of CHPG (500 μM) in CCI mice.

(d) Kymographs and △F/F traces of cells show Ca^{2+} signals evoked by bath application of CHPG in GCaMP7b^{+} astrocytes from CCI mice, which were blocked by mGluR5 antagonist MPEP in the presence of TTX and picrotoxin. The red line represents the mean and the shading represents ± SEM.

(f, g) Summary plots illustrate that Ca^{2+} signals elicited by CHPG are attenuated by mGluR5 antagonist MPEP **(f: n=17 cells from 4 mice; baseline (gray circle) = -0.09311 △F/F, CHPG (brownish green circle) = 0.01480 △F/F, *p*=0.0012, Paired t-test;** g: **baseline (gray circle) = -0.1110 △F/F, CHPG (brownish green circle) = -0.1129 △F/F, *p*=0.9075, Paired t-test)**. The empty circles represent the median.

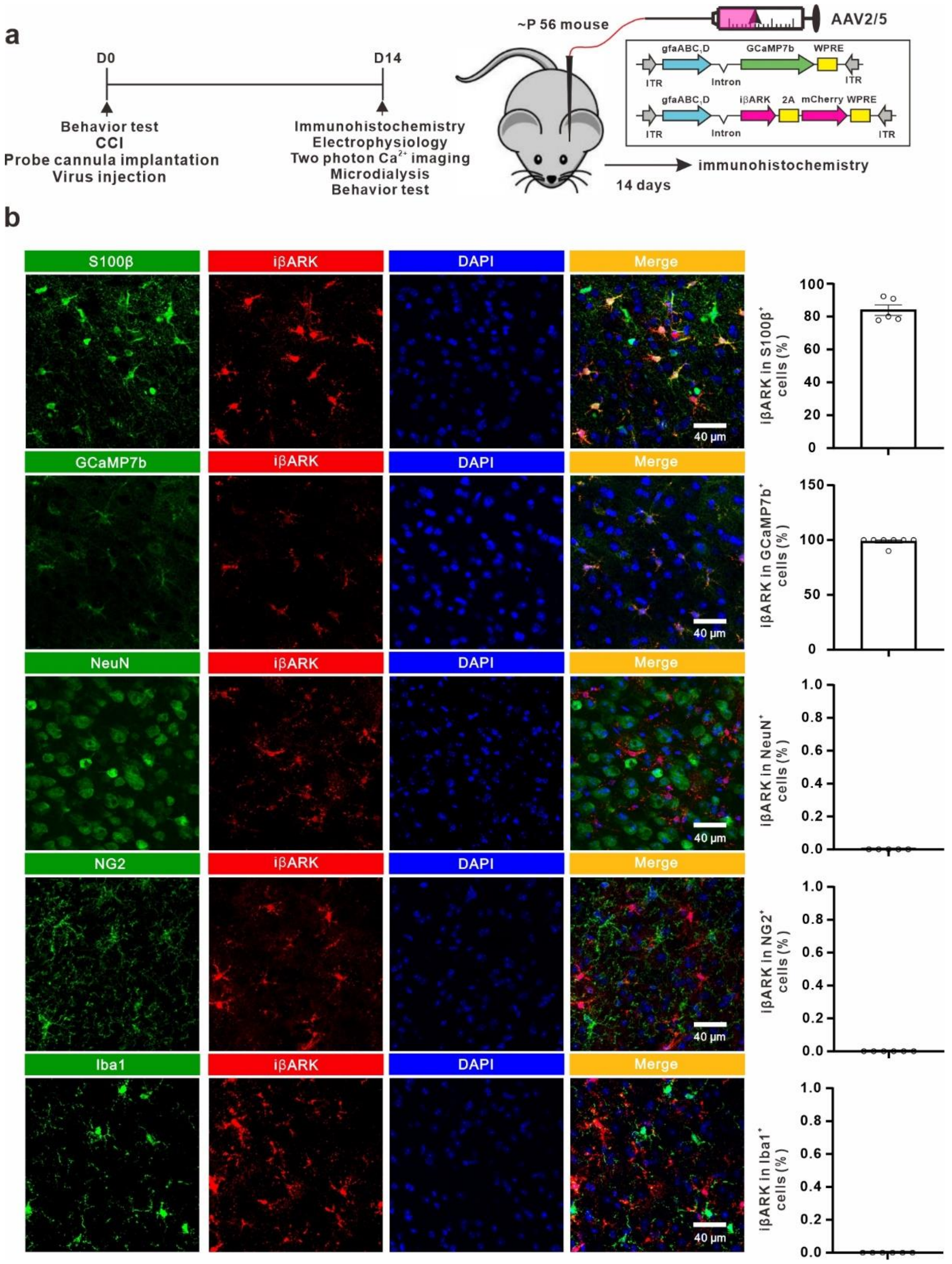

Supplementary Figure 3: iβARK specifically expressed in astrocytes in ACC.

(a) Schematic representation of the experimental design.

(b) Colocalization of iβARK (red) with the astrocytic-specific marker

s100β (green) **(83.91±3.169%, n=5 slices from 3 mice)** or GCaMP7b (green) in the ACC **(98.57±1.429%, n=7 slices from 3 mice)**, with no overlap with the neuronal marker NeuN (green) **(0.00±0.00%, n=5 slices from 2 mice)**, microglia specific marker Iba1 (green) **(0.00±0.00%, n=6 slices from 3 mice)** and oligodendrocyte precursor cell specific marker NG2 (green) **(0.00±0.00%, n=6 slices from 3 mice)**. Error bars represent the mean ± SEM.

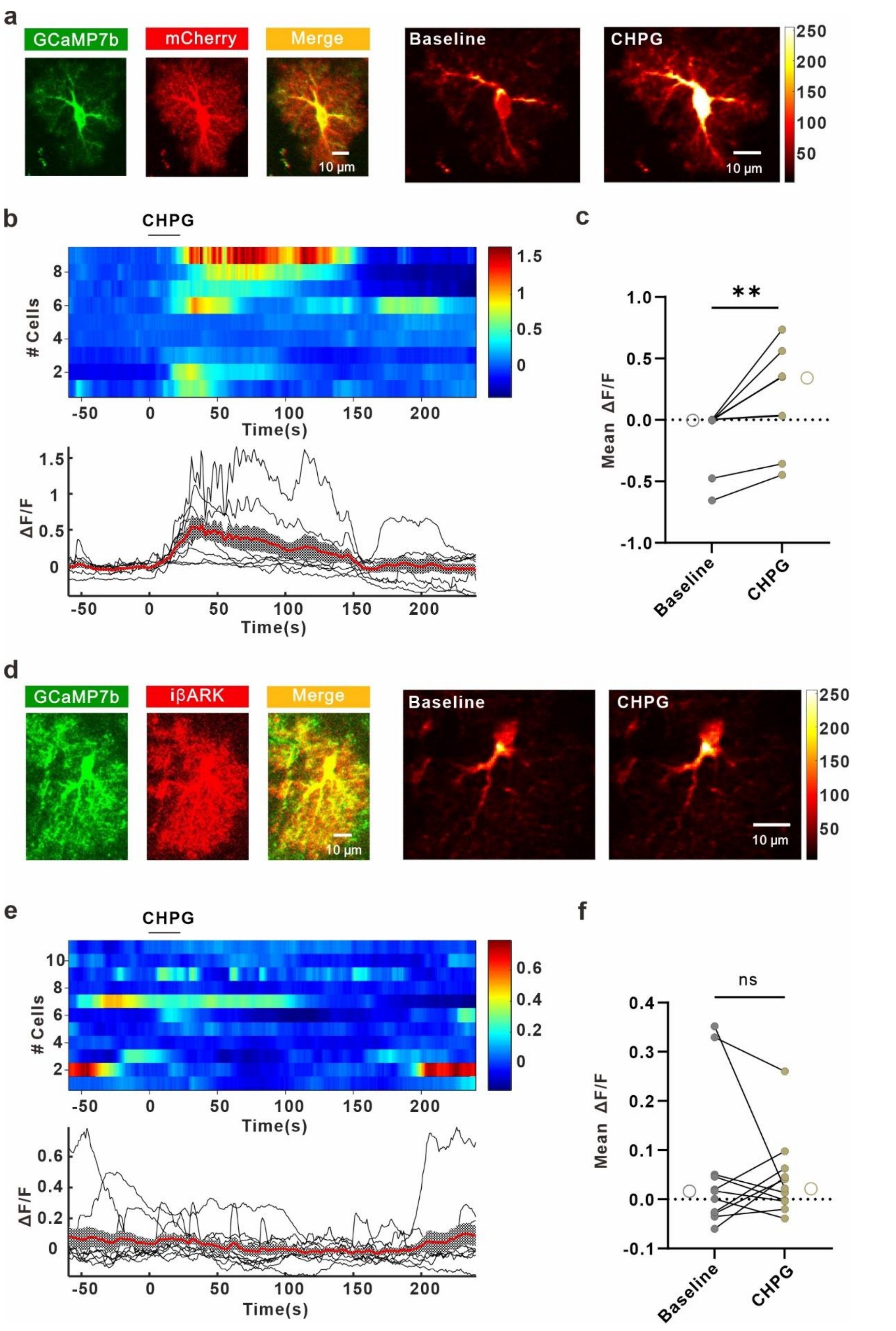

Supplementary Figure 4: iβARK attenuates mGluR5-evoked Ca^{2+} signaling in ACC in neuropathic pain mice.

(a) Representative two-photon images demonstrating the co-expression of GCaMP7b (green) and mCherry (red) constructs. Images depict a GCaMP7b$^+$ astrocyte before (left) and after (right) the application of CHPG (500 μM).

(b) Kymographs and △F/F traces of cells showing Ca^{2+} signals evoked by bath application of CHPG in GCaMP7b$^+$ astrocytes from mCherry-expressing mice in the presence of TTX and picrotoxin. The red line represents the mean and the shading represents ± SEM.

(c) Summary plots illustrating that astrocyte expression of mCherry has no effects on Ca^{2+} signals elicited by CHPG **(n=9 cells from 3 mice; baseline (gray circle) =0.001168 △F/F, CHPG (brownish green circle) =0.3488 △F/F, *p*=0.0048, Paired t-test)**. The empty circles represent the median.

(d) Representative two-photon images demonstrating the co-expression of GCaMP7b (green) and iβARK (red) constructs. Images depict a GCaMP7b$^+$ astrocyte before (left) and after (right) the application of CHPG (500 μM).

(e) Kymographs and △F/F traces of cells showing Ca^{2+} signals evoked by the bath application of CHPG in GCaMP7b$^+$ astrocytes from iβARK-expressing mice when in the presence of TTX and picrotoxin. The shading represents ± SEM. The red line represents the mean and the shading represents ± SEM.

(f) Summary plots illustrating that Ca^{2+} signals elicited by CHPG are

attenuated by expression of iβARK **(n=11 cells from 4 mice; baseline (gray circle) =0.01707 △F/F, CHPG (brownish green circle) =0.02205 △F/F, *p*=0.6514, Paired t-test)**. The empty circles represent the median.

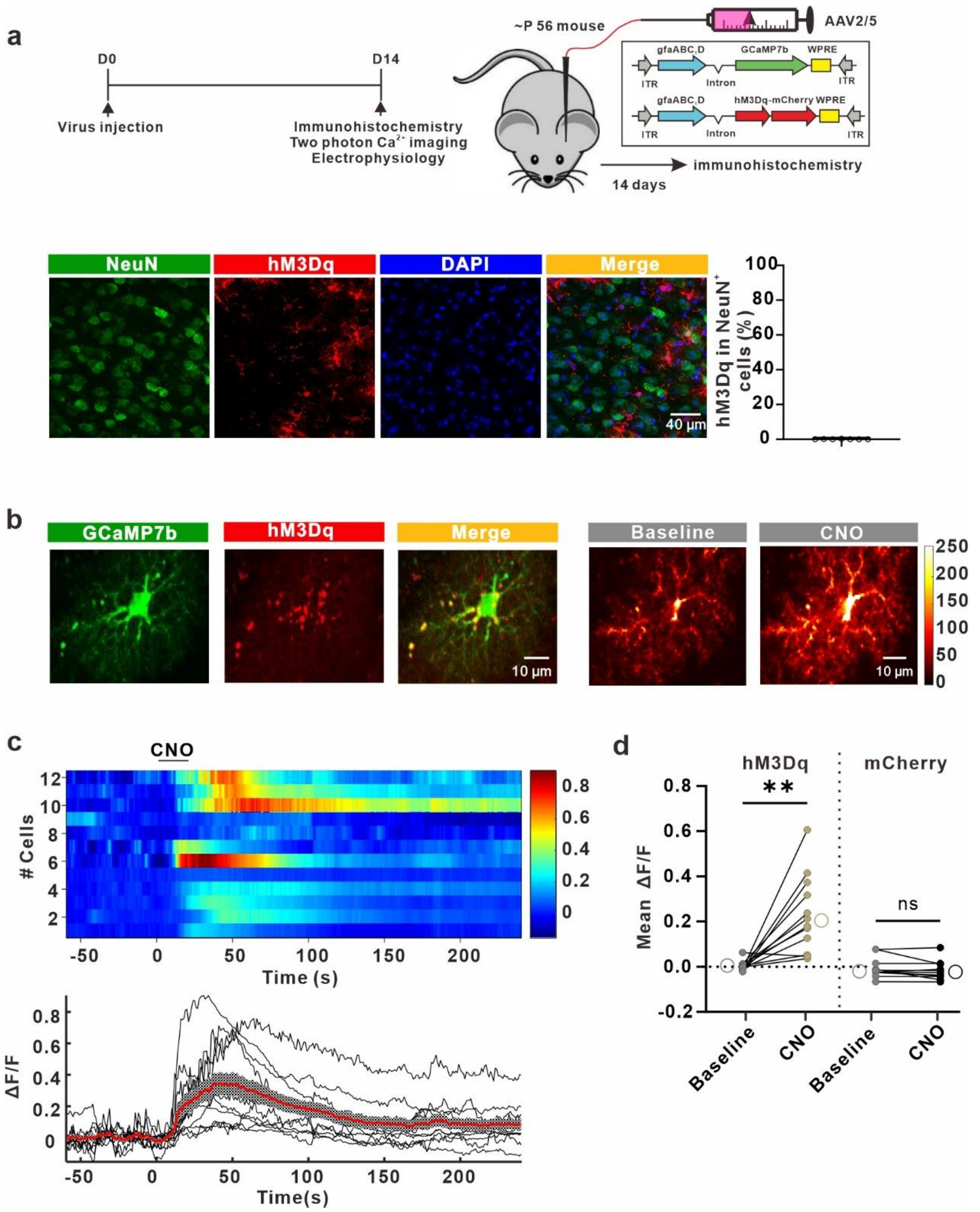

Supplementary Figure 5: CNO induces astrocytic Ca^{2+} signals in ACC.

(a) Illustration showing that hM3Dq (red) rarely overlap with the neuronal marker NeuN (green) **(0±0%, n=7 slices from 3 mice)**. Error bars represent the mean ± SEM.

(b) Representative two-photon images demonstrating the co-expression of

GCaMP7b (green) and hM3Dq (red) constructs. Images depict a jGCaMP7b+ astrocyte before (left) and after (right) the application of CNO (5 μM).

(c) Kymographs and △F/F traces of cells with Ca^{2+} signals evoked by the bath application of CNO in GCaMP67b+ astrocyte when in the presence of TTX and picrotoxin. The red line represents the mean and the shading represents ± SEM.

(d) Summary plot illustrating that CNO is effective to activate astrocytes in hM3Dq mice **(n=12 cells from 3 mice; baseline (gray circle) =-2.735e-006 △F/F, CNO (brownish green circle) =0.1958 △F/F, *p*=0.0012, paired t-test)**; however, astrocyte expressing only mCherry show no change in Ca^{2+} signals in response to CNO **(n=11 cells from 3 mice; baseline (gray circle) = -0.02610 △F/F, CNO (dark circle) = -0.02896 △F/F, *p*=0.1458, paired t-test)**. The empty circles represent the median.
