# Supplementary material for: mGluR5-mediated astrocytes hyperactivity in the anterior cingulate cortex contributes to neuropathic pain in male mice
Source: Commun Biol. 2025 Feb 20;8:266. doi: 10.1038/s42003-025-07733-5 (PMC11842833; doi:10.1038/s42003-025-07733-5)
Supplement: Supplementary file 3 — Description of Additional Supplementary Files [file 42003_2025_7733_MOESM3_ESM.docx]

**Description of Additional Supplementary File**

File name: Supplementary Data 1

Description: Source data – Numerical source data for all graphs in the manuscript can be found in supplementary data 1 file.
