# Supplementary material for: mGluR5-mediated astrocytes hyperactivity in the anterior cingulate cortex contributes to neuropathic pain in male mice
Source: Commun Biol. 2025 Feb 20;8:266. doi: 10.1038/s42003-025-07733-5 (PMC11842833; doi:10.1038/s42003-025-07733-5)
Supplement: Supplementary file 5 — Reporting summary [file 42003_2025_7733_MOESM5_ESM.pdf]

nature portfolio

Corresponding author(s): Weida SHEN

Last updated by author(s): Nov 14, 2024

Reporting Summary

Nature Portfolio wishes to improve the reproducibility of the work that we publish. This form provides structure for consistency and transparency in reporting. For further information on Nature Portfolio policies, see our Editorial Policies and the Editorial Policy Checklist.

Statistics

For all statistical analyses, confirm that the following items are present in the figure legend, table legend, main text, or Methods section.

n/a	Confirmed	
☐	☒	The exact sample size (*n*) for each experimental group/condition, given as a discrete number and unit of measurement
☐	☒	A statement on whether measurements were taken from distinct samples or whether the same sample was measured repeatedly
☐	☒	The statistical test(s) used AND whether they are one- or two-sided<br>*Only common tests should be described solely by name; describe more complex techniques in the Methods section.*
☒	☐	A description of all covariates tested
☐	☒	A description of any assumptions or corrections, such as tests of normality and adjustment for multiple comparisons
☐	☒	A full description of the statistical parameters including central tendency (e.g. means) or other basic estimates (e.g. regression coefficient) AND variation (e.g. standard deviation) or associated estimates of uncertainty (e.g. confidence intervals)
☐	☒	For null hypothesis testing, the test statistic (e.g. *F*, *t*, *r*) with confidence intervals, effect sizes, degrees of freedom and *P* value noted<br>*Give P values as exact values whenever suitable.*
☒	☐	For Bayesian analysis, information on the choice of priors and Markov chain Monte Carlo settings
☒	☐	For hierarchical and complex designs, identification of the appropriate level for tests and full reporting of outcomes
☒	☐	Estimates of effect sizes (e.g. Cohen's *d*, Pearson's *r*), indicating how they were calculated

Our web collection on statistics for biologists contains articles on many of the points above.

Software and code

Policy information about availability of computer code

Data collection	Commercial softwares licensed by microscopy companies was utilized: Olympus VT1000,FV31S-SW), Zeiss (LSM 980).<br>Digitized analog signals at 20 kHz were obtained with Digidata (version 1550B) and pClamp (version 10.6) software.
Data analysis	Calcium image analysis was performed with Image J version: 1.54f) and Astrocyte Quantification and Analysis(AQuA). We used Clampfit 10.6 to analyze the frequency and amplitude of mEPSCs, PPR.<br>Graph Pad Prism (version 10.0) was used for statistical analysis.

For manuscripts utilizing custom algorithms or software that are central to the research but not yet described in published literature, software must be made available to editors and reviewers. We strongly encourage code deposition in a community repository (e.g. GitHub). See the Nature Portfolio guidelines for submitting code & software for further information.

Data

Policy information about availability of data

All manuscripts must include a data availability statement. This statement should provide the following information, where applicable:

- Accession codes, unique identifiers, or web links for publicly available datasets
- A description of any restrictions on data availability
- For clinical datasets or third party data, please ensure that the statement adheres to our policy

Data availability statement:
Data are available from the corresponding author on reasonable request.

Human research participants

Policy information about studies involving human research participants and Sex and Gender in Research.

Reporting on sex and gender	This information was not collected as no human was involved in this study.
Population characteristics	This information was not collected as no human was involved in this study.
Recruitment	This information was not collected as no human was involved in this study.
Ethics oversight	This information was not collected as no human was involved in this study.

Note that full information on the approval of the study protocol must also be provided in the manuscript.

Field-specific reporting

Please select the one below that is the best fit for your research. If you are not sure, read the appropriate sections before making your selection.

☒ Life sciences ☐ Behavioural & social sciences ☐ Ecological, evolutionary & environmental sciences

For a reference copy of the document with all sections, see nature.com/documents/nr-reporting-summary-flat.pdf

Life sciences study design

All studies must disclose on these points even when the disclosure is negative.

Sample size	Pilot experiments, as well as previous work in our laboratories, were used to predict effect size and indicated n=5-10 per group to be sufficient to detect biologically and statistically significant results. No sample size calculation was performed.
Data exclusions	No data were excluded from the data set.
Replication	The data were repeated on multiple independent animals, all replicates are true biological (rather than technical) replicates. All replication were successful.
Randomization	Animals were randomly allocated to control or treatment groups.
Blinding	The investigators were blinded to group allocation during behavior test.

Reporting for specific materials, systems and methods

We require information from authors about some types of materials, experimental systems and methods used in many studies. Here, indicate whether each material, system or method listed is relevant to your study. If you are not sure if a list item applies to your research, read the appropriate section before selecting a response.

Materials & experimental systems

n/a	Involved in the study	
☐	☒	Antibodies
☒	☐	Eukaryotic cell lines
☒	☐	Palaeontology and archaeology
☐	☒	Animals and other organisms
☒	☐	Clinical data
☒	☐	Dual use research of concern

Methods

n/a	Involved in the study	
☒	☐	ChIP-seq
☒	☐	Flow cytometry
☒	☐	MRI-based neuroimaging

Antibodies

Antibodies used	For immunohistochemistry,we used anti-mouse s100β antibody(#287111,Synaptic Systems),anti-mouse NeuN antibody(#MAB377,Millipore),anti-rabbit mGluR5 antibody(#55920,Cell Signaling Technology),anti-rabbit Iba1 antibody(#019-19741,FUJIFILM Wako Pure Chemical Corporation),anti-rabbit NG2 antibody(#AB5320,Millipore).
Validation	Validation imformation of each antibody is as follows:anti-mouse s100β antibody(Raponi,Eric et al,Glia.55,2(2007):165-77.doi:10.1002/glia.20445),anti-mouse NeuN antibody(Liu,Yajun et al,BMC physiology.9 17.22 Sep.2009,doi:10.1186/1472-6793-9-17),anti-rabbit mGluR5 antibody(Goniotaki,Despoina et al.PLoS pathogens.13,11 e1006733.27 Nov.2017,doi:10.1371/journal.ppat.1006733),anti-rabbit(Sasaki,Y et al.research communications.286,2(2001):292-7.doi:10.1006/bbrc.2001.5388),anti-rabbit NG2 antibody(Kuboyama,Kazuya et al.PloS one.7,11(2012):e48797,doi:10.1371/journal.pone.0048797).

Animals and other research organisms

Policy information about studies involving animals; ARRIVE guidelines recommended for reporting animal research, and Sex and Gender in Research

Laboratory animals	Male C57BL/6 mice (~2 months old) were purchased from Hangzhou Ziyuan Laboratory Animal Corporation and were housed in groups of three to four per cage, with softened paper for enrichment in cages (35 cm in length, 13 cm in width, and 13 cm in height). The mice were maintained on a 12-hour light/dark cycle and given unrestricted access to food and water.
Wild animals	No wild animals were used in this study.
Reporting on sex	Only male mice were used in this study to avoid potential variability in pain sensitivity associated with the estrous cycle in females, which could influence the results of pain threshold measurements.
Field-collected samples	The study involved no samples collected from the field.
Ethics oversight	Our study was conducted in accordance with the Guide for the Care and Use of Laboratory Animals and received approval from the ethics committee of Hangzhou City University (registration number: 22061).

Note that full information on the approval of the study protocol must also be provided in the manuscript.
